# Biological role of Semaphorin 6D in the proliferation, migration and invasion of gastric cancer

**DOI:** 10.4314/ahs.v24i3.21

**Published:** 2024-09

**Authors:** Huan Zhou, Guang Chen

**Affiliations:** 1 Department of Medical Oncology, Second Affiliated Hospital of Dalian Medical University, Dalian, China; 2 Department of Traumatic Orthopedics, Second Affiliated Hospital of Dalian Medical University, Dalian, China

**Keywords:** Gastric cancer, extracellular signal-regulated kinase, semaphorin 6D, ERK and PI3K/AKT/mTOR, cell proliferation

## Abstract

**Background:**

To analyse how Semaphorin 6D (SEMA6D) expression and extracellular signal-regulated kinase (ERK), phosphatidylinositol 3-kinase/protein kinase B/mammalian target of rapamycin (PI3K/AKT/mTOR) signal pathways are activated and how they influence gastric cancer proliferation, migration, and invasion.

**Methodology:**

SEMA6D expression was knocked down in human gastric cancer cells using RNA interference technology. In vitro assays were used to analyse how SEMA6D knockdown affects clone formation, migration, and invasion. ERK and PI3K/AKT/mTOR signaling pathway related proteins were detected by immunoblotting.

**Results:**

In the si-NC group, Sema6D protein levels were higher than those in the si-1 and si-2 groups after knockdown of Sema6D, while in the si-1 group, Sema6D protein levels were higher than those in the si-2 group (P<0.05). P-PI3K, ERK/p-ERK, AKT/p-AKT, and mTOR/p-mTOR levels in the si-NC group were significantly higher than those in the si-1 and si-2 groups after knockdown of Sema6D (P < 0.05). It was found that scratch healing rate of SGC-7901 cells in si-NC group was higher than that in si-1 and si-2 groups, and the difference was statistically significant (P < 0.05).

**Conclusion:**

SEMA6D expression level can affect the biological behavior of gastric cancer cells.

## Introduction

Gastric cancer is a malignant tumor affecting the digestive system, and incidence is higher in developing countries than in developed countries. Gastric cancer in China accounts for about 42% of the world, and coastal areas are high-incidence areas of gastric cancer in China^1^. With the in-depth study of clinical gastric cancer, the critical role played by semaphorin (Sema) in tumor development has attracted clinical attention. Some studies^2^,^3^ have confirmed that semaphorin 6D (Sema6D) is associated with the proliferation and migration of various cancer cells, vascular regeneration, immune metabolism, and neural regeneration. As reported by some authors^4^,^5^, Sema6D can affect the activation of extracellular signal-regulated kinase (ERK) and phosphatidylinositol 3-kinase/protein kinase B/mammalian target of rapamycin (PI3K/AKT/mTOR) signaling pathways, which influences tumorigenesis, cancer cell proliferation, poor prognosis, and drug resistance. Given this, we speculate that the Sema6D expression level may affect the activation level of ERK and PI3K/AKT/mTOR signaling pathways in gastric cancer patients, thus affecting the proliferation and migration ability of gastric cancer cells. That down-regulation of Sema6D expression level can weaken ERK and PI3K/AKT/mTOR signaling pathways and thus inhibit gastric cancer cell proliferation, migration, and invasion.

## Materials and methods

### Cell lines

Human gastric cancer cell line SGC-7901 was purchased from American Type Culture Collection (ATCC), and human gastric epithelial cell GES-1 was purchased from Shanghai Zhibai Biotechnology Co., Ltd. (Shanghai, China).

### Instruments and reagents

The laboratory equipment listed includes a desktop highspeed centrifuge, a pipette made by Eppendorf in Hamburg, Germany, an ECL luminescence imager, a Bio-Rad RT-PCR instrument from Hercules, CA, USA, a light microscope and an inverted microscope from Olympus in Tokyo, Japan. Also included are a CO2 incubator from Panasonic in Osaka, Japan, a thermostatic refrigerator made by Haier in China, a thermostatic water bath, a liquid nitrogen tank, a shaker, a microelectronic balance from Beijing Taizerida Technology Co., Ltd. in Beijing, China, an ultraviolet (UV) spectrophotometer, a protein electrophoresis apparatus, a cell incubator and a cell bench from Thermo in Waltham, MA, USA. In addition, the laboratory uses various reagents and kits, such as RIPA lysate, TBST rinse, Transwell 1640, trypsin, RPMI 1640 from Corning in Corning, NY, USA, SYBR fluorescent dye, reverse transcription kit from TaKaRa in Tokyo, Japan, OPTI-MEM medium, Lipofectamine 3000, PCR primers, HRP-labeled goat anti-rabbit IgG, polyvinylidene fluoride membrane, protein quantification kit (BCA method), TRIzol reagent from Invitrogen in Carlsbad, CA, USA, fetal bovine serum (FBS), RPMI1640 medium from Zhongqiao Xinzhou in Shanghai, China, chemiluminescence reagent, and an immunohistochemical kit from Millipore in Billerica, MA, USA. Finally, the laboratory also uses various antibodies for experiments, including anti-GAPDH/AKT/p-AKT/mTOR and anti-P-PI3K/ERK/p-ERK/p-mTOR from Wuhan Sanying Biotechnology Co., Ltd. in Wuhan, China, and anti-Sema6D from CST in Danvers, MA, USA.

## Methods

### Cell culture and Sema6D protein expression level detection

To detect the proliferation ability of SGC-7901 cells, a plate cloning assay was performed. Firstly, the cells were digested into a cell suspension and centrifuged at 800 rpm for 3 minutes. After removing the supernatant, the cells were resuspended in culture medium, thoroughly mixed and then 10 µL of the cell suspension was injected into a counting plate using a pipette. The SGC-7901 cells were diluted to 5 × 105 cells/mL and counted using a cell counter. Then, 500 cells were inoculated into each well of a 6-well plate, shaken well, and incubated overnight. Once the number of cells per colony was greater than 50, the culture medium was removed and the cells were washed twice with PBS. The cells were then fixed with 5% polymethanol and stained with Giemsa staining solution for 15 minutes at room temperature. After washing off the floating color, photos were taken and the number of colony formations were analysed using Image-J software.

### Knockdown of Sema6D expression by si-RNA synthesis versus transfection

Human gastric cancer cells SGC-7901 with good growth status were selected for the experiment, SGC-7901 cells were added into RPMI1640 + 10% FBS medium, 5% CO_2_ and cultured at 37°C at a constant temperature, and the cells were diluted to 1 × 10^6^ cells/mL overnight before transfection, and then inoculated into a 6-well plate with fusion ≥ 5 0% before transfection (Lipofectamine 3000), Cells were collected after 24 h and 48 to extract RNA and protein, respectively, and divided into a si-NC group (3′-5′: ACGUGACACGUUCGGAGAATT; 5′-3′: UUUCUCCGAACGUGUCACGUTT), si-1group (3′-5′: UAUGUCUGUAAUAGACUGCTT; 5′-3′: GCAGUCUAUUGACAUATT), and si-2 group; the si-NC group was the control group, si-1 was knockdown Sema6D, the si-2 group was further knockdown Sema6D based on si-1 group.

### SGC-7901 Cell Proliferation Assay

The plate cloning assay was used to detect the proliferation ability of SGC-7901 cells, digest the cells into cell suspension, centrifuge at 800 rpm for 3 min and 800 rpm, remove the supernatant, add culture medium to resuspend the cells, thoroughly blow and mix the cells, inject 10 µL of the mixed cell suspension into the counting plate using a pipette, insert into the cell counter, dilute the SGC-7901 cells to 5 × 10^5^ cells/mL, use the cell counter for counting, inoculate the SGC-7901 cells into a 6-well plate at a number of 500 cells/well, shake well and place in an incubator for overnight, remove the culture medium to terminate the culture when the number of cells is > 50/colony, wash twice with PBS, fix the room temperature and stain with 5% polymethanol and Giemsa staining solution for 15 min, respectively, wash off the floating color and take photos, and analyse the number of colony formation with Image-J software.

### Detection of migration and invasion of SGC-7901 cells

Transwell assay and scratch healing assay were used to detect the migration and invasion of SGC-7901 cells. SGC-7901 cells in the logarithmic growth phase were taken and digested into cell suspension. After centrifugation at 800 rpm for 3 min, the supernatant was removed, and serum-free medium was added to resuspend the cells. The density of SGC-7901 cells was calculated and adjusted to 8 × 10^5^ cells/mL. Then 100 cell suspension and 700 complete medium (containing 10% FBS) were added to the upper and lower chambers of the transwell, and the cells were cultured at 5% CO^2^ and 37°C for 24 h. After the completion of the culture, the inserts were removed and washed. 4% paraformaldehyde and 0.5% crystal violet staining solution were used to fix the constant temperature and staining for 15 min, respectively, and the cells were dried after washing. In the invasion assay, in addition to preparing Matrigel gel in the upper chamber of the transwell. The rest of the operations were identical to the migration assay. Cell scratch healing assay: SGC-7901 cells in the logarithmic growth phase were digested into cell suspension, centrifuged at 800 rpm for 3 min, and the supernatant was removed. Serum-free medium was added to resuspend the cells. SGC-7901 cells were diluted to 1 × 10^6^ cells/mL using a cell counter, and the cells were thoroughly mixed by blowing, then inoculated in a 6-well plate, shaken well, and the pipette was used to draw a vertical line when the cell confluence was 100%. During the scratch process, the tip was kept perpendicular to the 6-well plate, and then the exfoliated cells were washed off using PBS. The scratch healing of the cells at 0 h and 24 h was observed and photographed. The scratch healing rate of the cells was calculated to be (S1-S2)/S1 × 100%, of which S1 and S2 indicated the area at 0 h and 24 h, respectively; the migration and invasion ability of SGC-7901 cells were analysed.

### Detection of protein expression levels related to ERK and PI3K/AKT/mTOR signaling pathway

SGC-7901 cells transfected for 48 h were collected, and treated with RIPA lysis solution to extract total protein, and the protein concentration was measured using a BCA protein quantification kit; 25 of complete protein was pipetted for Western blotting experiments, and the protein was transferred to a polyvinylidene fluoride membrane and blocked with 5% skimmed milk at room temperature for 1.5 h. anti-GAPDH/AKT/p-AKT/mTOR and anti-P-PI3K/ERK/p-ERK/p-mTOR at 1:1000 were added, respectively, p-PI3K, ERK/p-ERK, AKT/p-AKT, mTOR/p-mTOR protein expression was measured in three groups of SGC-7901 cells, and gray values of ERK and PI3K/AKT/mTOR signaling pathway-related proteins were analysed by Image-J software.

### Statistical analysis

Graphpad Prism 8.0 (La Jolla, CA, USA) and Statistical Product and Service Solutions (SPSS) 22.0 software (IBM, Armonk, NY, USA) were used to analyse the data. Image-J software analysed the gray values of proliferation ability and protein bands related to ERK and PI3K/AKT/mTOR signaling pathways in SGC-7901 cells. One-way analysis of variance was used to compare the measurement data between multiple groups. T-test for independent samples was used to compare the measurement data between the two groups. A chi-square test was applied to the categorical information. P < 0.05 was statistically significant.

## Results

### Sema6D protein expression in gastric cancer cells and gastric epithelial cells

**Figure 1:**
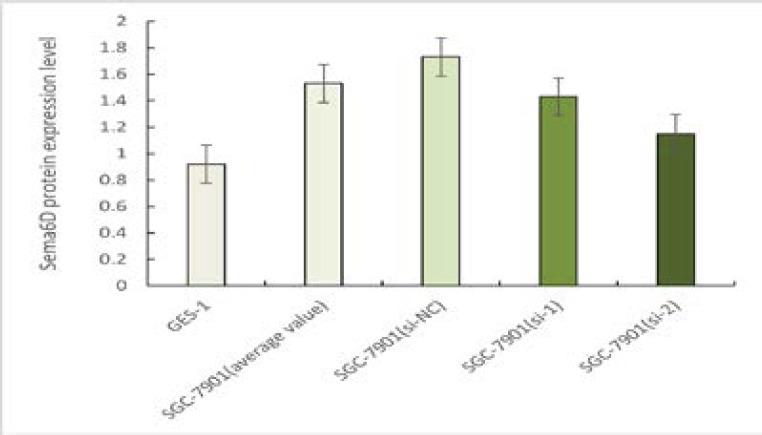
Sema6D protein expression level in SGC-7901 and GES-1 cell

The Sema6D protein assay results showed that the Sema6D protein level in human gastric cancer cells SGC-7901 (mean of si-NC, si-1, si-2 groups) was higher than that in human gastric epithelial cells GES-1, and the difference was statistically significant (*P* < 0.05). Comparison of Sema6D protein levels in SGC-7901 among three groups of human gastric cancer cells showed that Sema6D levels in the si-NC group were higher than those in the si-1 and si-2 groups after knockdown Sema6D, and Sema6D protein levels in the si-1 group were higher than those in the si-2 group. The differences were statistically significant (*P* < 0.05), as shown in Figure 1.

### Effect of Sema6D knockdown on ERK and PI3K/AKT/mTOR signaling pathway protein expression

**Figure 2:**
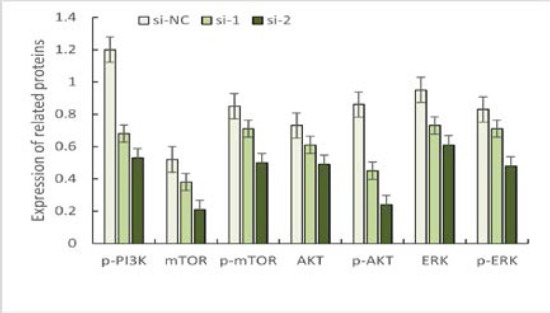
Expression of ERK and PI3K/AKT/mTOR signaling pathway proteins in the three groups

The results of downstream signaling pathway-related protein detection showed that the knockdown of Sema6D was able to down-regulate p-PI3K, ERK/p-ERK, AKT/p-AKT, mTOR/p-mTOR, and other protein expression levels. Compared among the three groups, the levels of p-PI3K, ERK/p-ERK, AKT/p-AKT, and mTOR/p-mTOR in the si-NC group were higher than those in the si-1 and si-2 groups after Sema6D knockdown, and the levels of p-PI3K, ERK/p-ERK, AKT/p-AKT, mTOR/p-mTOR in the si-1 group were higher than those in the si-2 group. The differences were statistically significant (*P* < 0.05), as shown in Figure 2.

### Effect of Sema6D knockdown on SGC-7901 cell proliferation

**Figure 3:**
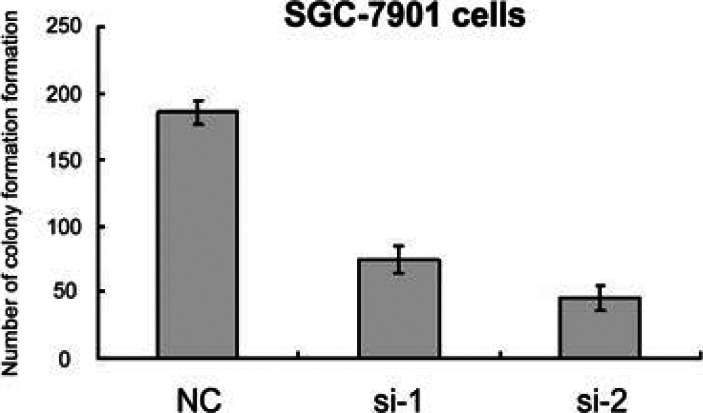
The Cell Proliferation Numbers of SGC-7901 cells in three groups

The SGC-7901 cell proliferation assay results showed that the knockdown of Sema6D could inhibit SGC-7901 cell proliferation. The number of colony formation formations of SGC-7901 cells in the si-NC group was greater than that in the si-1 group and si-2 group. The number of colony formations in the si-1 group was greater than that in the si-2 group. Te difference was statistically significant (*P* < 0.05), as shown in Figure 3.

### Effect of Sema6D knockdown on migration and invasion of SGC-7901 cells

**Figure 4:**
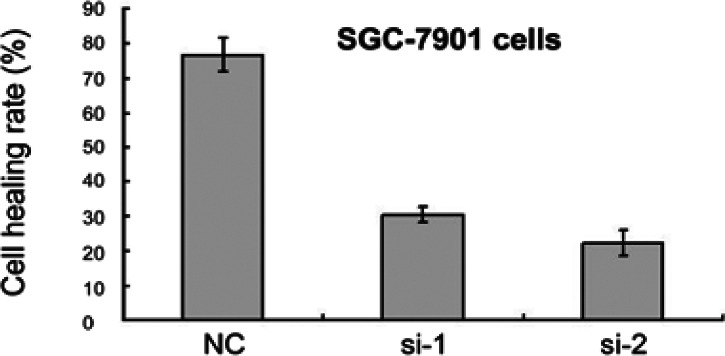
The Migration and invasion rate of three groups SGC-7901 cells

The results of the SGC-7901 cell migration and invasion assay showed that knockdown of Sema6D could inhibit SGC-7901 cell migration and invasion. The scratch healing rate of SGC-7901 cells in the si-NC group was higher than that in the si-1 group and si-2 group, and the scratch healing rate in the si-1 group was higher than that in the si-2 group, and the difference was statistically significant (*P* < 0.05), as shown in Figure 4.

## Discussion

In clinical practice, gastric cancer is a common malignant tumor of the digestive system. The progression of gastric cancer is slow. Symptoms are often absent in the early stages of the disease. The growth of cancer will cause abdominal discomfort, unexplained diarrhea, melena, peritoneal irritation, and other symptoms, all of which are harmful and fatal^6^-^8^. Gastric cancer is a complex disease, and clinical studies^9^,^10^ have shown that gastrointestinal tumors, polyps, inflammation, genetics and radiation all play an important role. SEMAs are most transmembrane proteins with similar physicochemical structure and function and play an essential physiological role in immune regulation, tumor growth, angiogenesis, immune regulation, and neurological function^11^.

As part of the SEMA family, Sema6D comes in four forms, of which Sema6D is known to be associated with the development and occurrence of non-small cell lung cancer, gastric cancer, and osteoma. In addition to being a SEMA protein^12^,^13^ that has been extensively studied in clinical settings, SEMA6D may also mediate the development and occurrence of these benign and malignant tumors. A study by Sixuan et al.^14^ found that SEMA6D levels were significantly higher in cancer tissues from patients with primary gastric cancer than in adjacent gastric mucosa tissues, similar to the results of this study. The study also demonstrated that patients with gastric cancer with deeper cancer invasion, worse differentiation, and higher clinical stage had higher Sema6D levels in their cancer tissues. The study by Hughes et al.^15^ showed that Sema6D could affect the activation status of ERK and PI3K/AKT/mTOR signaling pathways and the expression levels of downstream related proteins in humans, thus affecting the proliferation and invasion of tumor cells.

As part of this study, the human gastric cancer cell line SGC-7901 and the human gastric epithelial cell line GES-1 were used to knock down SEMA6D expression in SGC-7901 cells using siRNA synthesis and transfection techniques. Based on the degree of Sema6D knockdown, SGC-7901 cells were divided into three groups: si-NC, si-1, and si-2. Plate cloning, immunoblotting, transwell, and cell scratch healing assays were used to analyse the effect of SEMA6D expression levels on SGC-7901 cell colony formation ability, activation status of ERK and PI3K/AKT/mTOR signaling pathways, and migration and invasion. The results showed that the knockdown of Sema6D could inhibit SGC-7901 cell colony formation, and the higher the degree of knockdown, the lower the number of SGC-7901 cell colony formations. The results also showed p-PI3K, ERK/p-ERK, AKT/p-AKT, and mTOR/p-mTOR in the si-2 group after knocking down Sema6D were lower than those in the si-NC group and si-1 group. At the same time, the scratch healing rate in the si-2 group was also lower than that in the si-NC group and si-1 group, i.e., Knockdown of Sema6D could down-regulate p-PI3K, ERK/p-ERK, AKT/p-AKT, mTOR/p-mTOR, and other protein expression levels in gastric cancer tissues, thereby inhibiting cancer cell migration and invasion. In this case, ERK, PI3K/AKT/mTOR signaling is closely related to tumor cell signaling, where they function as signal transducers and scaffolds, play a role in the conversion and regulation of integrin adhesion sites in tumor cells, and then promote cancer cell migration, proliferation, survival, and invasion. In addition to its physiological role in immune regulation, tumorigenesis, development, angiogenesis, immunity, and neurological regulation, Sema6D signaling pathways include ERK, PI3K/AKT/mTOR, and others. As a result of silencing Sema6D in gastric cancer patients, ERK signaling pathway protein expression was reduced, inhibiting the proliferation, migration, and invasion of gastric cancer cells^16^,^17^.

## Conclusion

In summary, the Sema6D expression level can affect the biological behavior of gastric cancer cells. Knockdown of Sema6D can down-regulate ERK, PI3K/AKT/mTOR signaling pathway-related protein expression and inhibit the proliferation, migration and invasion of gastric cancer cells, which is expected to be an effective target for gastric cancer drug development and has clinical research value.

## Data Availability

The datasets used and analysed during the current study are available from the corresponding author on reasonable request.
